# Diagnostic Yield of Universal Urine Toxicology Screening in an Unselected Cohort of Stroke Patients

**DOI:** 10.1371/journal.pone.0144772

**Published:** 2015-12-16

**Authors:** Rizwan Kalani, Eric M. Liotta, Shyam Prabhakaran

**Affiliations:** 1 Department of Neurology, University of Washington, Seattle, Washington, United States of America; 2 Department of Neurology, Northwestern University, Chicago, Illinois, United States of America; Federico II University of Naples, ITALY

## Abstract

**Background:**

Illicit drug use increases the risk of cerebrovascular events by a variety of mechanisms. A recent report suggested that universal urine toxicology (UTox) screening of patients with stroke may be warranted. We aimed to evaluate the diagnostic yield of urine drug screening among unselected patients admitted with acute stroke or transient ischemic attack (TIA).

**Methods:**

Using a single-center prospective study design, we evaluated consecutive patients with acute ischemic stroke, TIA, intracerebral hemorrhage (ICH), or subarachnoid hemorrhage (SAH) over one year. Urine samples were collected within 48 hours of admission and analyzed for common classes of abused drugs. Prevalence of positive UTox screening was determined. We evaluated whether baseline demographics and clinical factors were associated with UTox results.

**Results:**

Of 483 eligible patients (acute ischemic stroke 66.4%; TIA 18.8%; ICH 7.7%; SAH 7.0%), 414 (85.7%) completed UTox screening. The mean (standard deviation) age was 65.1 (15.6) years, 52.7% were male, and 64.3% were Caucasian. Twenty-two (4.6%) patients had positive screening—cannabinoids were detected in 13 cases (3.1%), cocaine in 5 cases (1.2%), amphetamines in 1 case, and phencyclidine in 1 case. The highest yield (14.1%) was observed in patients < 60 years old with history of tobacco use while it was < 5% in the remaining subgroups (p<0.01).

**Conclusions:**

Consistent with current guidelines, a selective approach to UTox screening should be pursued in acute stroke evaluation. The highest diagnostic yield is likely to be for cannabinoids and cocaine testing in younger patients with a history of concurrent tobacco use.

## Introduction

According to the National Survey on Drug Use and Health, an estimated 23.9 million Americans ages 12 and older used illicit drugs in 2012 [1]. The Global Burden of Disease Study estimates that illicit drug dependence accounted for 20 million disability adjusted life-years worldwide in 2010 [2].

Both psychomotor stimulants and psychotomimetic drugs have been associated with stroke. Cocaine increases the risk of both cerebral infarction and hemorrhage through a variety of mechanisms [3]. A more recent case-control study suggests an association between cannabis use and ischemic stroke [4]. All forms of amphetamine use have been associated with stroke, and, though limited to case reports, phencyclidine use has been linked to intracerebral hemorrhage [5–7]. Although the prevalence of cocaine use worldwide has declined in recent years, cannabis and amphetamine use have been increasing [8].

The current American Stroke Association/American Heart Association guidelines for early management of acute ischemic stroke recommend that toxicology screens should be used in selected cases [9] such as in evaluation of stroke in younger patients in whom the etiology is otherwise not evident. A recent study by Silver *et al*. argued for universal screening on the basis that 11% of patients with stroke or transient ischemic attack (TIA) tested positive for cocaine [10]. However, the demographics of the population studied may not be representative of other institutions and only selected patients completed toxicology screening, potentially introducing biases [10]. In this study, we aimed to evaluate the diagnostic yield of unselected drug screening among patients admitted with acute stroke or TIA at a tertiary care medical center.

## Materials and Methods

### Study Cohort

This study was approved by the Northwestern University institutional review board. Using a single-center prospective study design, consecutive patients ≥ 18 years with a diagnosis of acute ischemic stroke/TIA, intracerebral hemorrhage (ICH), or subarachnoid hemorrhage (SAH) were consented and enrolled in the Northwestern University Brain Attack Registry. Written informed consent was obtained from the patient or their legally authorized representative. Criteria for diagnosis of stroke and TIA were based on recent recommendations and final diagnosis is adjudicated by board-certified vascular neurologists [11]. We queried the registries to identify all enrolled patients from August 1, 2012 to July 31, 2013. Patients were included if they 1) were admitted to the stroke unit or neurosciences intensive care unit (NSICU); and 2) had urine toxicology screening within 48 hours of admission, the reported retention times of drugs in urine [12]. We prospectively captured patient age, race, sex, vascular risk factors, current tobacco use, urine toxicology screen results, initial National Institutes of Health Stroke Scale (NIHSS) scores, and ischemic stroke subtype by TOAST (Trial of Org 10172 in Acute Stroke Treatment) classification [13].

### Drug Screen

Urine samples of at least 10 mL from a single void were collected on admission to the inpatient stroke unit or NSICU and sent for qualitative testing for common classes of abused drugs. Screening was done by enzyme multiplied immunoassay technique (EMIT II, Siemens Healthcare Diagnostics, Deerfield, IL, USA). This assay has demonstrated high sensitivity and specificity for detection of substances evaluated [14]. We defined presence of cocaine, amphetamines, cannabinoids, and/or phencyclidine as positive screens as these have all been linked to stroke mechanism.

### Statistical Analysis

Patient characteristics were described as means/standard deviations or medians/interquartile ranges for continuous variables and proportions for categorical variables. We estimated the prevalence of having any positive urine toxicology screen and also individual elements (i.e. cocaine, cannabis) and various combinations and used the Wald method to estimate 95% confidence intervals around a point estimate. We evaluated associations between demographics, clinical factors, and stroke subtype and urine toxicology results using Chi-square or Fisher’s exact tests for categorical variables and student t-tests or Mann-Whitney tests for continuous variables, as appropriate. A p-value < 0.05 was considered significant. All analyses were conducted with SPSS (IBM Corp. Released 2012, Version 21.0. Armonk, NY).

## Results

Of 483 patients admitted with acute stroke or TIA, 414 (85.7%) completed urine toxicology evaluations within 48 hours of admission. This was the final cohort of patients analyzed in our study. The remaining 69 patients did not complete the toxicology screen during their hospital course. In a sensitivity analysis, patients completing toxicology screening were less likely to have prior stroke or TIA (20.0% vs 31.9%, p = 0.027), less likely to have ICH (7.7% vs. 25.0%, p < 0.01), and more likely to acknowledge tobacco use (43.5% vs. 29.0%, p = 0.024) than those who did not undergo screening; there was no significant differences in demographics (age, sex, race-ethnicity), other vascular risk factors, or baseline NIHSS score.

Among patients completing urinary toxicology screening, the mean age (±SD) was 65.1 ± 15.6 years and 52.7% were male and 64.3% were Caucasian. Final diagnosis was ischemic stroke in 66.4%, TIA in 18.8%, ICH in 7.7%, and SAH in 7.0%. The distribution of TOAST classification of patients with ischemic stroke/TIA was as follows: 21.0% cardioembolic, 18.1% large artery atherosclerosis, 15.0% small vessel disease, 9.9% other determined, and 36.0% cryptogenic.

Cocaine, cannabinoids, amphetamines, or phencyclidine were detected in 19 (4.6%, 95% CI 3.0–7.1) patients. Screening for cannabinoids was positive in 13 total cases (3.1%) and cocaine was detected in 5 (1.2%) individuals. One patient tested positive for amphetamines and was taking a prescribed medication accounting for the toxicology result. Phencyclidine was also detected in a single patient.

**Fig 1:**
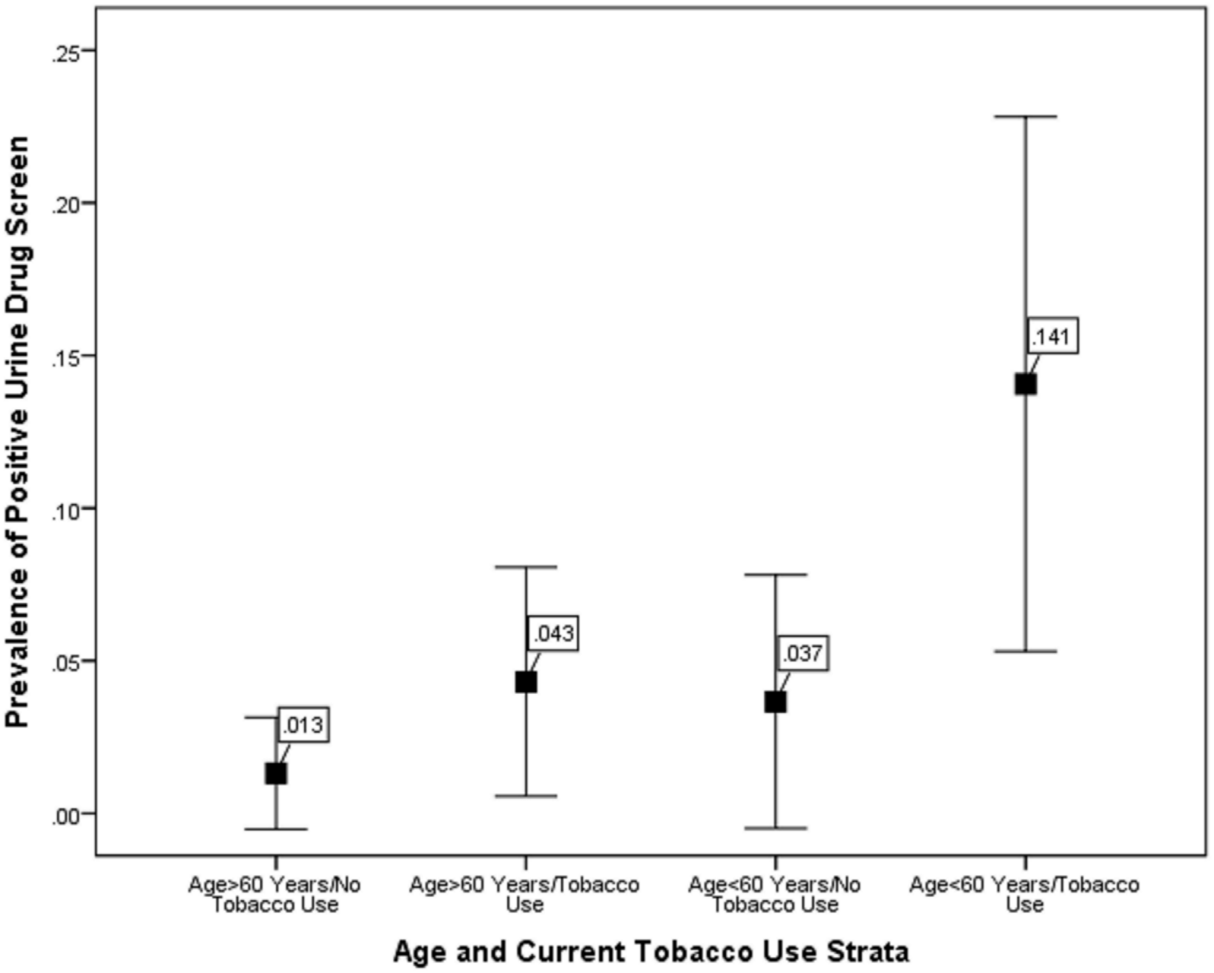
Prevalence and 95% confidence intervals for positive urine drug screen by age and tobacco use strata (p<0.001).

Table 1 compares demographics, vascular risk factors, admission NIHSS score, and final diagnosis between patients with positive and negative drug screens. Patients with positive urine toxicology screening were significantly more likely to be younger and report current tobacco use. Combining these 2 variables into 4 strata (age ≥ 60 years/no tobacco use; age < 60 years/no tobacco use; age ≥ 60 years/tobacco use; age < 60 years/tobacco use), the prevalence of positive urine drug screen increased across strata (p < 0.01; Fig 1) from 1.3% (95% 0.4–4.7) to 14.1% (95% CI 7.6–24.6).

**Table 1:** Comparison of baseline demographics, risk factors, and stroke characteristics among those with positive vs. negative urine toxicology screens.

	Urine Toxicology Positive (n = 19)	Urine Toxicology Negative (n = 395)	P-value
Demographics			
Age, years (mean ± SD)	57.6 ± 11.3	65.5 ± 15.7	0.032
Age < 60 years, n (%)	12 (63.2)	134 (33.9)	0.009
Male, n (%)	12 (63.2)	206 (52.2)	0.348
Race, n (%)			
Caucasian	12 (63.2)	254/295 (64.3)	0.603
African-American	7 (36.8)	112 (28.4)	
Other	0 (0)	29 (7.3)	
Vascular Risk Factors, n (%)			
Hypertension	11 (57.9)	296 (74.9)	0.097
Diabetes	3 (15.8)	87 (22.0)	0.520
Atrial fibrillation/flutter	1 (5.3)	48 (12.2)	0.364
Prior stroke or TIA	6 (31.6)	77 (19.5)	0.199
Coronary artery disease	4 (21.1)	65 (16.5)	0.599
Tobacco use	14 (73.7)	166 (42.0)	0.007
Clinical Features			
Median NIHSS (IQR)	4.0 (1.0–8.0)	2.0 (0.0–5.0)	0.133
Stroke type			
Ischemic stroke	15 (78.9)	260 (65.8)	0.695
TIA	2 (10.5)	76 (19.2)	
Intracerebral hemorrhage	1 (5.3)	31 (7.8)	
Subarachnoid hemorrhage	1 (5.3)	28 (7.1)	

SD = standard deviation, IQR = interquartile range, NIHSS = national institute of health stroke scale, TIA = transient ischemic attack

## Discussion

In a consecutive series of patients admitted to a stroke unit at a single tertiary care medical center, we found that the yield of urine toxicology screening for a substance that has been associated with an increased risk of stroke was only 4.6%. Cannabinoids and cocaine were the principal drugs detected. Current smokers under 60 years of age had the highest likelihood of a positive toxicology result (14.1%). Our study suggests that universal screening in patients with stroke or TIA is likely to be associated with low diagnostic yield and selected approaches may be more reasonable.

Multiple mechanisms of stroke have been related to illicit drug use. Cocaine ingestion may cause ischemic stroke through vasospasm, by preventing sympathomimetic neurotransmitters and cerebral vasoconstrictors reuptake [3, 15]. It may also result in acute myocardial infarction and arrhythmia, or cardiomyopathy with subsequent cerebral embolism [16]. Cannabinoids, particularly marijuana may induce reversible cerebral vasoconstriction syndrome and multifocal arterial stenosis, which may cause cerebral ischemia [17, 18]. Proposed mechanisms of ischemic stroke with amphetamine exposure include intracranial cerebral necrotizing vasculitis, non-arteritic vasculopathy, as well as possible cardiomyopathy with subsequent cerebral embolism [5, 19, 20]. The mechanism of ischemic stroke in relation to phencyclidine use is unclear. It should be noted that stroke in the setting of illicit drug use may not be secondary to the effects of acute exposure and traditional mechanisms should not be overlooked.

Several differences may explain our lower yield (1.2% vs. 11% cocaine-positive screens) compared to a prior study [10] and compared to the population prevalence’s of drug use. The racial/ethnic background of the population evaluated in the previous report differed in comparison to the cohort in our study, which may contribute to risk of drug use. In addition, the highest rate of cocaine positive toxicology in the prior study was in patients with hemorrhagic stroke as opposed to those with ischemic stroke or TIA [10]. However, selection bias such that only 40% of the total cohort completed toxicology screening may have resulted in the higher prevalence compared to our study where 86% of all eligible patients underwent screening. While population lifetime prevalence of any illicit drug abuse is approximately 9%, the rate of drug use restricted by type to those associated with stroke and ingested within 48 of stroke is likely much lower. Similar to their findings and consistent with prior reports, however, we also observed a higher risk of illicit drug use in younger populations with stroke [21, 22]. No prior study has demonstrated an association of tobacco use and detection of illicit drug use in patients with cerebrovascular events. Based on these risk factors and contrary to the prior paper suggesting universal testing, our data support a targeted or selective approach to urine toxicology screen in stroke patients.

Given the concerning trends in healthcare spending and costly diagnostic evaluation of patients with stroke, testing should be limited when yield is low [23]. Though it may vary between institutions, a single urine toxicology screen at some centers may exceed 300 US Dollars. An approach based on pretest probability and clinical context seems to be the most appropriate strategy for allocating health care resources. It is also not advisable to screen at time periods after which illicit drugs cannot be detected by current techniques. Unlike cocaine and cannabis use, testing for amphetamines and phencyclidine is likely to be of very low yield in cerebrovascular ischemia, though could be considered if their use is more common in the local practice setting or in patients with hemorrhagic stroke.

There are some limitations to our study. First, despite a protocol of performing urine toxicology screening universally on patient admission to the stroke unit or NSICU, 14.3% did not complete testing, introducing the potential for some selection bias. Reasons for non-adherence to protocol included: failure to obtain an adequate urine sample from the patient, testing not appropriately ordered or cancelled, or the sample being lost. Second, our toxicology screening does not differentiate distinct types of substances within each tested drug class (e.g., dextroamphetamine vs. methylphenidate). Third, since illicit drug use can result in systemic organ involvement (i.e., myocardial ischemia) in addition to cerebrovascular complications, some patients with concomitant cardiac symptoms may have been admitted to non-stroke units and not included in our registry. Lastly, it is theoretically possible that obtaining a urine sample immediately after substance use and stroke symptom onset could have led to false negative urine toxicology screens in certain cases.

In conclusion, our study supports the current recommendation that selective urine toxicology screening be considered in stroke and TIA [9]. The highest diagnostic yield is likely to be for cannabinoids and cocaine screening, in younger patients with a history of concurrent tobacco use. Local epidemiology, particularly patient demographics, should be considered along with these predictive factors in the decision for testing. Determining exposure to drugs of abuse, by history or diagnostic screening, would allow for early detection and opportunity for counseling and patient education regarding the association of substance abuse and stroke. Further studies are warranted to evaluate overall diagnostic yield of toxicology screening in different populations, for newly emerging illicit drugs, and for cost effectiveness of testing.

## Abbreviations

UTox: urine toxicology
TIA: transient ischemic attack
ICH: intracerebral hemorrhage
SAH: subarachnoid hemorrhage
NSICU: neurosciences intensive care unit
TOAST: Trial of Org 10172 in Acute Stroke Treatment
